# UNDEREXPLORED SOLUTIONS IN DETROIT'S NEIGHBORHOODS: THE IMPORTANCE OF LEGACY TO ADDRESS ENVIRONMENTAL CONCERNS

**DOI:** 10.1093/geroni/igac059.1093

**Published:** 2022-12-20

**Authors:** Tam Perry, Evan Villeneuve, Brenda Butler, Fatima Hazimeh, Ventra Asana

**Affiliations:** Wayne State University, Detroit, Michigan, United States; ClearCORPS Detroit, Detroit, Michigan, United States; Detroit's Eastside Community Network/Marlowe Stoudamire Wellness Hub, Detroit, Michigan, United States; Wayne State University, Dearborn, Michigan, United States; Independent Scholar, Detroit, Michigan, United States

## Abstract

Neighborhood infrastructure challenges and changes are possibly understood and experienced in unique ways across the lifespan. This study presents findings on neighborhood conditions and underexplored solutions from the perspective of older adults in Detroit, Michigan. This project obtained multiple perspectives on these issues from older Detroiters through interviews (n-19) and professionals working on climate concerns in the region (n=5). The research was designed using community-based research approaches including having older adults as members of the research team from instrument design to dissemination. One emergent theme, the importance of legacy as motivation in addressing environmental concerns, will be highlighted. This presentation will conclude with a discussion of next steps for this work.

